# Glucagon-Like Peptide-1(9-36) Inhibits Chemokine-Induced Migration of Human CD4-Positive Lymphocytes

**DOI:** 10.1371/journal.pone.0058445

**Published:** 2013-03-04

**Authors:** Ana Liberman, Melanie Esser, Nikolaus Marx, Mathias Burgmaier

**Affiliations:** Department of Internal Medicine I – Cardiology, University Hospital Aachen, Aachen, Germany; University of Padova, Italy

## Abstract

**Introduction:**

Inhibitors of dipeptidyl peptidase-IV (DPP-IV), which decrease the degradation of glucose-lowering GLP-1(7-36) to the metabolically inactive GLP-1(9-36), are current new treatment options for patients with type 2 diabetes mellitus, a high-risk population for cardiovascular disease. However, the effects of the metabolite GLP-1(9-36) on atherosclerosis are unknown. Thus, the present study examined the effect of GLP-1(9-36) on chemokine-induced CD4-positive lymphocyte migration as one of the early and critical steps in atherogenesis.

**Methods and Results:**

Stimulation of isolated human CD4-positive lymphocytes with SDF-1 led to a 3.4 fold (p<0.001; n = 7) increase in cell migration. Pretreatment of cells with GLP-1(9-36) reduced this effect in a concentration-dependent manner by 41% to a 2.0 fold induction at 10 nmol/L GLP-1(9-36) (p<0.001 compared to SDF-1-treated cells, n = 7). Similar effects were obtained when RANTES was used as a chemokine to induce cell migration. The action of GLP-1(9-36) on CD4-positive lymphocyte migration was mediated through an early inhibition of chemokine-induced PI-3 kinase activity. Downstream in the PI-3 kinase signaling pathway, GLP-1(9-36) inhibited SDF-1-induced phosphorylation of MLC and cofilin and decreased f-actin formation as well as ICAM3 translocation as shown by Western blotting, flow cytometry and immunohistochemistry, respectively. However, the effect of GLP-1(9-36) on PI-3 kinase signaling was not associated with increased intracellular levels of cAMP. Furthermore, experiments with siRNA demonstrated that the inhibitory effect of GLP-1(9-36) on SDF-1-induced ICAM3-translocation was preserved in human CD4-positive lymphocytes lacking the GLP-1 receptor, suggesting signaling independent of the known GLP-1 receptor.

**Conclusion:**

Thus, GLP-1(9-36) inhibits chemokine-induced CD4-positive lymphocyte migration by inhibition of the PI3-kinase pathway independent of cAMP and GLP-1 receptor signaling. Further studies are needed to assess whether such effects may be clinically relevant for patients with type 2 diabetes treated with DPP-IV inhibitors.

## Introduction

Glucagon-like peptide-1 (GLP-1) analoga and inhibitors of dipeptidyl peptidase-IV (DPP-IV) are current new treatment options for patients with type 2 diabetes mellitus, a high-risk population for atherosclerosis [1], [2], [3]. Both drugs have distinct pharmacodynamics: GLP-1 analoga directly stimulate the GLP-1 receptor, whereas DPP-IV inhibitors act by slowing the rapid degradation of GLP-1(7-36) to GLP-1(9-36) [4], [5]. Therefore, beneficial vascular effects of the metabolite GLP-1(9-36) could be of significant clinical interest.

Whereas various effects of metabolically active GLP-1(7-36) on multiple organs have been suggested, only limited evidence exists about the actions of GLP-1(9-36) on the cardiovascular system (for review see [5]). Recently, a role of GLP-1(9-36) in preventing ischemia-reperfusion injury has been suggested in isolated perfused mouse hearts [6]. Work from another group showed that the degradation product GLP-1(9-36) improves left ventricular and systemic hemodynamics using a canine model of dilated cardiomyopathy [7]. We have previously demonstrated that GLP-1(1-37), a long and untruncated form of GLP-1, inhibits chemokine-induced CD4-positive lymphocytes migration, an early and critical step of atherogenesis [8], [9]. This effect was shown to be dependent on the GLP-1 receptor protein and exendin-4, a specific GLP-1 receptor agonist, had similar effects on T-cell migration. However, while GLP-1(7-36) is the physiologic agonist of the GLP-1 receptor and increases plasma concentrations of insulin after food intake, the metabolite GLP-1(9-36) is an antagonist of the GLP-1 receptor and metabolically inactive [10], [11]. The effects of the metabolite GLP-1(9-36) on human CD4-positive lymphocyte migration are unknown.

As patients with type 2 diabetes are a high-risk population for the development of a severe and diffuse atherosclerosis, the potential action of these drugs as modulators of vascular disease is of significant clinical interest. Given the different pharmacodynamics of GLP-1 analoga and DPP-IV inhibitors, it is crucial to better understand the effects of the degradation product GLP-1(9-36) on mechanisms involved in atherogenesis. Thus, we investigated the effects of GLP-1(9-36) on the chemokine-induced migration of human CD4-positive lymphocytes as an early and critical step in atherogenesis.

## Methods

### Cell Culture

Buffy coats were obtained completely anonymized from the Department of Transfusion Medicine, University Hospital of Aachen. As the buffy coats were obtained completely anonymized, no ethical approval was necessary for this study in Germany. Human CD4-positive lymphocytes were isolated from buffy coats as has been previously described [9], [12]. Briefly, a Ficoll-Histopaque (Sigma) gradient centrifugation was used to obtain mononuclear cells and subsequent negative selection of CD4-positive T-cells was performed by magnetic bead separation (Miltenyi Biotech). The purity of CD4-positive lymphocytes was >97% as determined by flow cytometry.

### In vitro Cell Migration Assay

For in vitro cell migration assays, human CD4-positive lymphocytes were cultured in serum-free media for 16 h. A 48-well microchemotaxis chamber (Neuroprobe) was used to investigate T-cell chemotaxis [9]. Wells in the upper and lower chamber were separated by a polyvinylpyrrolidone-free polycarbonate membrane (pore size 5 µm; Whatman). CD4-positive cells were pre-treated for 30 min with GLP-1(9-36) (Phoenix) at the concentrations indicated before 3 hours of incubation with SDF-1 (Upstate) or RANTES (PeproTech). Migrated cells attached to the bottom face of the filter, which had been pre-incubated with collagen, and were subsequently stained and counted under the light microscope. Furthermore, a scrambled GLP-1(9-36), which consists of the same amino acids as GLP-1(9-36) but in a random order (Val-Arg-Asp-Ile-Ala-Leu-Glu-Lys-Gly-Gly-Thr-Ala-Glu-Leu-Phe-Phe-Val-Tyr-Ala-Gly-Lys-Thr-Gln-Ser-Ser-Ser-Trp-Glu-NH3, Thermo Fisher Scientific) was included in the migration experiments as a negative control.

### Western Blot Analysis

Standard western blot analysis was performed using specific antibodies against p-cofilin, p-MLC (Cell Signaling) and human GLP-1 receptor (Upstate). Western blot images were analyzed by Carestream molecular imaging software (Carestream). For determination of protein levels, bands were normalized to ß-actin bands.

### Quantitative RT-PCR

RNA was extracted using the RNeasy extraction kit (Quiagen). cDNA was generated by reverse transcription (SuperScript reverse transcriptase, Invitrogen). Quantitative RT-PCR was performed using a commercially available Taqman Gene Expression Assay for the full length GLP-1 receptor (Applied Biosystems). Samples were normalized to a control gene (GAPDH, forward primer: 5′-GCC TCG TCC CGT AGA CAA AA-3′; reverse primer: 5′-TGG CAA CAA TCT CCA CTT TGC-3′).

### CXCR4 Expression and Vitality

Standard flow cytometry was performed using a CANTO II (BD) and a PE-labeled antibody against human CXCR4 (BD Pharmigen). For the determination of vitality, 1×10^6^ cells were treated with the same conditions as for the migration experiments and stained with trypan blue (Sigma Aldrich). Vital cells were counted in a Neubauer chamber.

### Phosphatidylinositol Kinase Assay

A PI-3 kinase activity assay was performed as has been previously described [12]. Briefly, human CD4-positive T-cells were incubated after isolation for 16 hours in RPMI medium without serum. Cells were pretreated for 30 min with and without GLP-1(9-36) and were then stimulated with 100 ng/mL SDF-1 for 5 min. The PI 3-kinase activity assays were performed using an goat anti-human p85 antibody (Santa Cruz) [13].

### f-actin Staining

For f-actin staining CD4-positive lymphocytes were pre-treated with 10 nmol/L GLP-1(9-36) for 30 min. Then, CD4-positive lymphocytes (3×10^6^ cells/mL) were treated with 100 ng/mL SDF-1 for times indicated. Cell stimulation was stopped by fixation using 3.7% (wt/vol) paraformaldehyde in PBS, pH 7.4. Cells were then washed at room temperature in TBS (50 mmol/L Tris-HCl, pH 7.6, 150 mmol/L NaCl, 0.1% NaN3). Cells were permeabilized with 0.1% Triton X-100 for intracellular staining of f-actin for 10 min. Cell suspensions were incubated for 30 min in PBS with FITC-conjugated phalloidin (Sigma). Flow cytometry analysis was performed in a FACScan cytofluorometer (Guave) and induction of f-actin was measured as previously described [12].

### ICAM3 Staining

To determine ICAM3 translocation, immunofluorescence staining for ICAM3 was performed on human CD4-positive lymphocytes as has been previously described [9], [13]. 3×10^5^ CD4 positive lymphocytes were incubated in 4-well plates (Costar Corp.) in a final volume of 1 ml complete medium on coverslips coated with collagen. Prior to treatment with SDF-1 (100 ng/mL, 30 min), cells were pre-incubated with GLP-1(9-36) for 30 min. CD4-positive lymphocytes were then fixated with 3.7% (wt/vol) paraformaldehyde in PBS and rinsed in TBS. Cells were incubated with a specific mouse anti-human ICAM3 antibody (BD Pharmingen) and after washing with PBS, an carboxymethylindocyanine 3-Cy3-coupled goat anti-mouse antibody (Jackson) was added for 30 min. After washing slides were counterstained with DAPI. Images were recorded with a fluorescence microscope (Leica) and cells in 5 randomly selected fields (∼ 100 cells) were analysed. If ICAM-3 was located evenly on the outer side of the plasma membrane, T-cells were considered to be resting. When a clear clustering of ICAM3 at the uropod was visible, ICAM3 translocation was considered to be present.

### cAMP Assay

For determination of intracellular cAMP concentrations 1×10^6^ isolated, human CD4-positive lymphocytes were treated with 10 nmol/L GLP-1(9-36) (Phoenix) or 100 ng/ml SDF-1 (Upstate) for times indicated. 100 ng/ml Forskolin (Calbiochem) was used as a positive control. The cAMP competitive ELISA (Thermo Scientific) was performed according to the manufactureŕs protocol. Briefly, cAMP was extracted by adding 0.1 mol/l HCl with 0.5% triton X-100 to the cells. After centrifugation at 600 g for 10 min, the supernatant was used for the determination of cAMP levels by competitive cAMP ELISA.

### Transfection with siRNA of the GLP-1 Receptor

Transfection of human CD4-positive lymphocytes with siRNA of the GLP-1 receptor has been performed as has been previously described [9]. Briefly, specific and negative siRNAs were purchased from Dharmacon. CD4-positive lymphocytes were isolated from blood. After isolation, cells were incubated for one hour in RPMI with 5% human serum. The human T-cell-nucleofector kit (Amaxa Biosystems) was used according to the manufacturer’s protocol employing 200 nM GLP-1 receptor siRNA. After transfection, cells were incubated in 12-well plates with RPMI containing 5% human serum for 48 hours. Untreated cells as well as cells treated with the amaxa nucleofector solution and negative siRNA were used as negative controls.

### Statistical Analysis

The data are reported as mean±standard error of the mean (SEM). Differences were analyzed by one- or two-way-ANOVA followed by the appropriate post-hoc test where applicable. A p-value <0.05 was regarded as statistically significant.

## Results

### GLP-1(9-36) Inhibits Chemokine-induced CD4-positive Lymphocyte Migration

**Figure 1:**
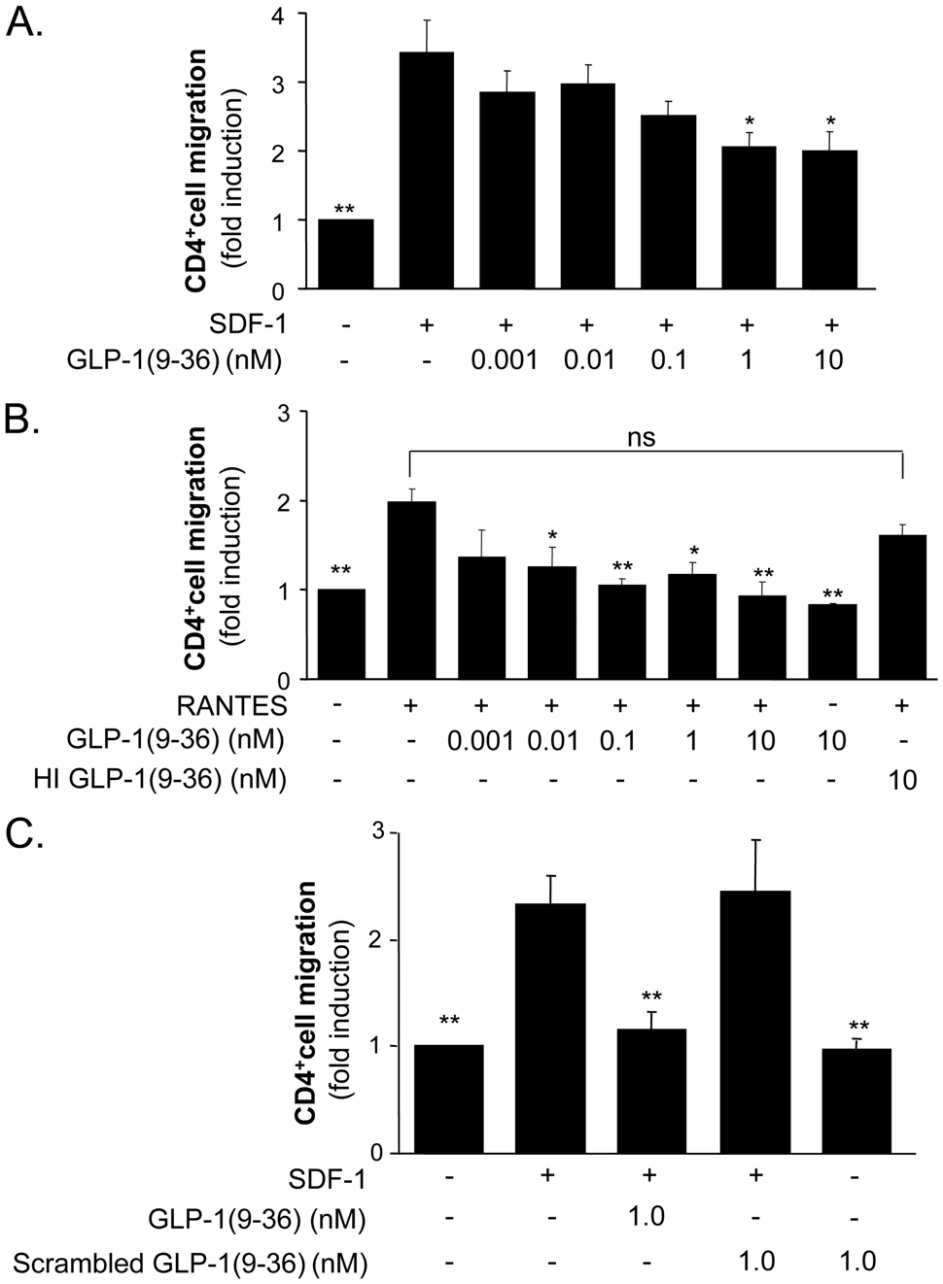
GLP-1(9-36) inhibits chemokine-induced migration of isolated human CD4-positive lymphocytes. A. and B.: Cells were pretreated with GLP-1(9-36) for 30 minutes at concentrations indicated before migration experiments using SDF-1 (100 ng/mL) (A) or RANTES (100 ng/mL) (B) were performed for three hours in the modified Boyden chamber. The data show that GLP-1(9-36) inhibits both SDF- and RANTES- induced migration of human CD4-positive lymphocytes. C: Scrambled GLP-1(9-36) does not reduce SDF-1-induced T-cell migration. Cells were pretreated with 1 nM GLP-1(9-36) and 1 nM scrambled GLP-1(9-36) for 30 minutes before migration experiments using SDF-1 (100 ng/mL) were performed for three hours in the modified Boyden chamber. Data are expressed as fold induction of unstimulated cells. Bars represent mean±SEM (n = 7); ***p<0.001, **p<0.01, *p<0.05 compared with SDF-stimulated cells.

To investigate the effect of GLP-1(9-36) on CD4-positive lymphocyte migration, experiments in the modified Boyden chamber were performed with isolated human CD4-positive lymphocytes. SDF-1 treatment increased cell migration by 3.4 fold (p<0.001; n = 7) and pretreatment of cells with GLP-1(9-36) reduced this effect in a concentration-dependent manner by 41% to a 2.0 fold induction at 10 nmol/L GLP-1(9-36) (p<0.001 compared with SDF-1 treated cells; n = 7) (Fig. 1A). Similar results were seen when RANTES was used as an inductor of CD4-positive lymphocyte migration (Fig. 1B) suggesting that the action of GLP-1(9-36) on T-cell migration is independent of the chemokine used.

Further migration experiments were performed to exclude that the phenomenon observed is caused by unspecific effects of a 28 amino acid peptide. In these experiments a scrambled GLP-1(9-36) was used, which consists of the same amino acids as GLP-1(9-36) but in a random order. Scrambled GLP-1(9-36) did not inhibit SDF-1-induced human CD4-positive lymphocyte migration (Fig. 1C). Neither scrambled GLP-1(9-36) nor native GLP-1(9-36) induced CD4-positive lymphocyte migration by themselves (Fig. 1B and 1C). Furthermore, heat-inactivated GLP-1(9-36) did not inhibit SDF-induced CD4-positive lymphocyte migration (Fig. 1B).

GLP-1(9-36) did not affect the expression of the chemokine receptor CXCR4 and did not influence cell viability as shown by flow cytometry and trypan blue staining, respectively (table 1).

**Table 1:** GLP-1 does not decrease the expression of the chemokine receptor CXCR4 and does not influence cell viability; n = 4; the data is displayed as mean ± SEM.

	Co	SDF-1	SDF-1+GLP-1(9–36)
		CXCR4 (normalized)	
mean	1	0.96	1.2
SEM	0	0.11	0.2
		Vitality (living cells out of 1 Million)	
mean	984 750	983 125	989 750
SEM	4 262.8	2 361.0	3 542.2

### GLP-1(9-36) Inhibits SDF-1-induced PI-3 Kinase Activity in Isolated Human CD4-positive Lymphocytes

**Figure 2:**
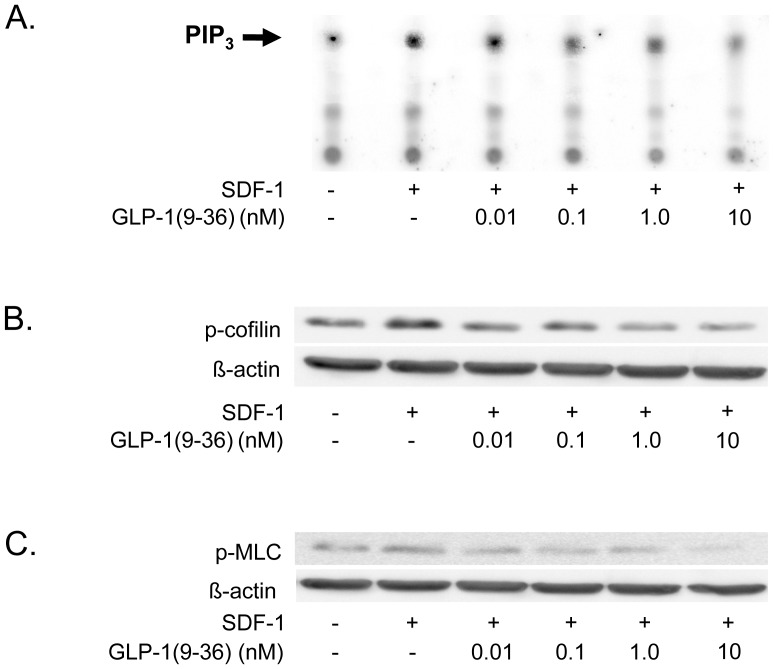
The PI3-kinase signaling cascade is involved in the inhibitory effect of GLP-1(9-36) on human lymphocyte migration. A: GLP-1(9-36) reduces SDF-1-induced PI-3 kinase activity in isolated human CD4-positive lymphocytes. Cells were pretreated with GLP-1(9-36) for 30 minutes at concentrations indicated before stimulation with SDF-1 (100 ng/mL) for 5 min. An arrow indicates PIP_3_. Three independent experiments showed similar results. B+C: GLP-1(9-36) inhibits SDF-1-induced phosphorylation of cofilin (B) and MLC (C) in isolated human CD4-positive lymphocytes. Cells were pretreated with GLP-1(9-36) for 30 min at concentrations indicated before stimulation with SDF-1 (100 ng/mL). Western blot analysis for p-cofilin and p-MLC was performed after 5 min. ß-actin protein expression served as a loading control. Representative results of 8 (p-cofilin) and 6 (p-MLC) independent experiments are shown.

Downstream of the chemokine receptor, the activation of PI-3 kinase is a crucial step in chemokine-induced CD4-positive lymphocyte migration [14]. Thus, an activity assay was performed to assess the action of GLP-1(9-36) on chemokine-induced PI-3 kinase activity. As demonstrated in figure 2A, GLP-1(9-36) significantly inhibited SDF-1-induced PI-3 kinase activity, suggesting that GLP-1(9-36) modulates an upstream step in the chemokine-activated signaling cascade in isolated human CD4-positive lymphocytes.

### GLP-1(9-36) Reduces SDF-1-induced Phosphorylation of Cofilin and MLC in CD4-positive Lymphocytes

Downstream in the PI3-kinase signaling cascade, chemokine stimulation of CD4-positive lymphocytes leads to serine phosphorylation of the enzymes cofilin and MLC. GLP-1(9-36) inhibited both SDF-1-induced phosphorylation of cofilin (fig. 2B) and MLC (fig. 2C) in a concentration-dependent manner, thus inhibiting additional processes downstream in the PI3-kinase signaling cascade.

### GLP-1(9-36) Reduces ICAM3 Translocation in Isolated Human CD4-positive Lymphocytes

**Figure 3:**
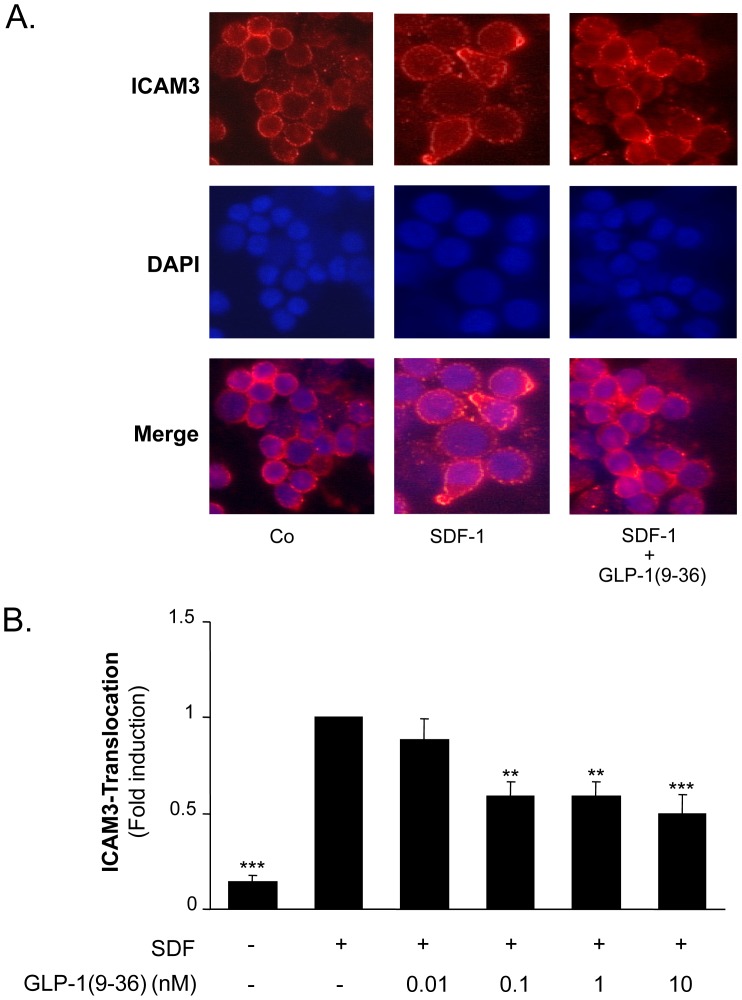
GLP-1(9-36) reduces SDF-1-induced ICAM3 translocation in isolated human CD4-positive lymphocytes. A.: Cells were pretreated with GLP-1(9-36) for 30 min at concentrations indicated before stimulation with SDF-1 for 30 min. ICAM3 translocation was determined using immunofluorescence staining. T-cells were considered to be resting if ICAM-3 was located evenly. If a clear clustering of ICAM3 at the uropod region was visible, ICAM3 translocation was considered to be present. B: Statistical analysis of cells positive for ICAM3 translocation as fraction of polarized cells; bars represent mean±SEM; n = 6; **p<0.01; ***p<0.001 compared with SDF-1-stimulated cells.

As ICAM3 translocation to the uropod of migrating CD4-positive lymphocytes is a crucial step in cell adhesion during migration [15], we next examined the effect of GLP-1(9-36) on ICAM3 translocation by immunofluorescence staining. As demonstrated in figure 3, GLP-1(9-36) significantly inhibited SDF-1-induced ICAM3 translocation to the uropod by 50.0% (p<0.001, n = 6).

### GLP-1(9-36) Inhibits f-actin Formation in CD4-positive Lymphocytes

**Figure 4:**
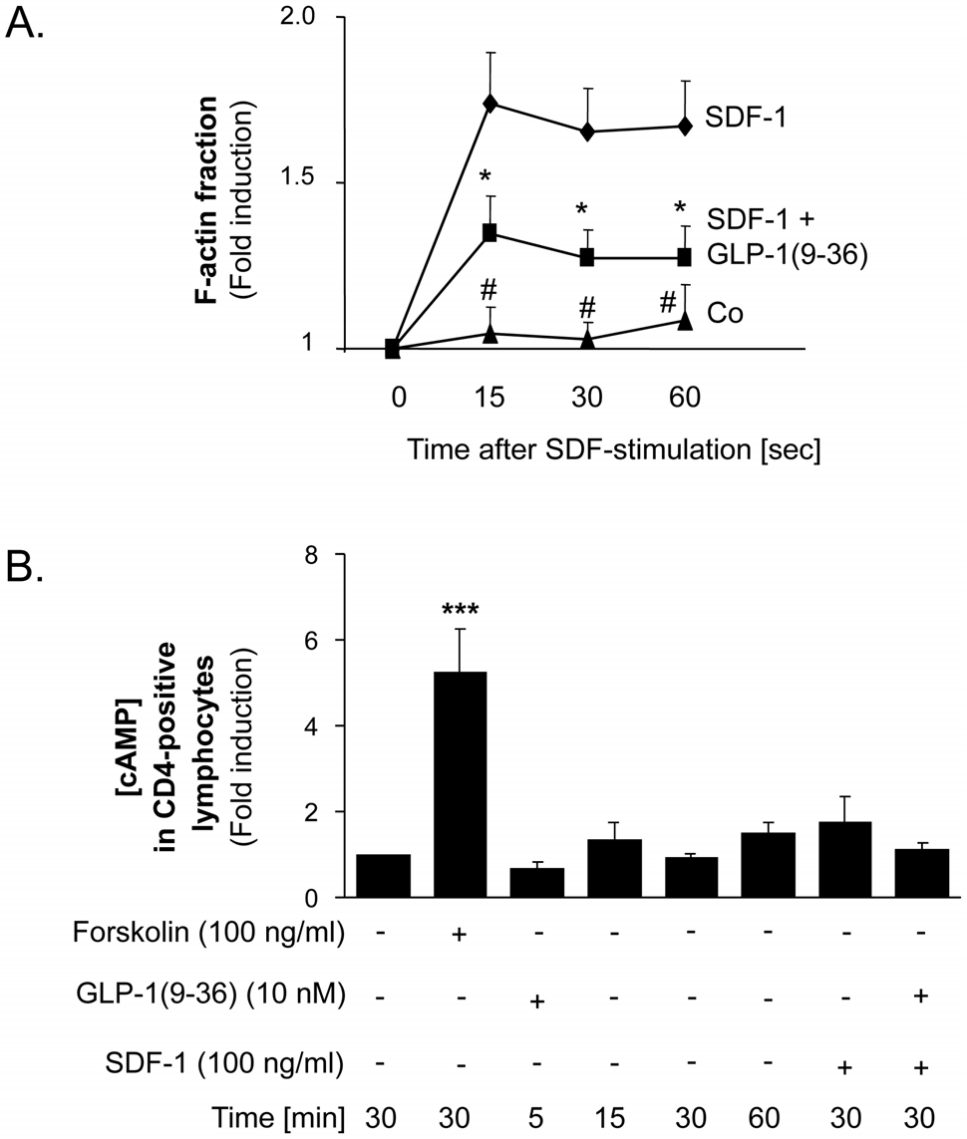
GLP-1 reduces f-actin formation but does not change intracellular cAMP-concentrations in isolated human CD4-positive lymphocytes. A: GLP-1(9-36) reduces SDF-1 induced f-actin formation in isolated human CD4-positive lymphocytes. Cells were pretreated with GLP-1 for 30 min (10 nmol/L) before stimulation with SDF-1. Actin polymerisation was assayed by flow cytometry at time points indicated. Dots represent mean. *p<0.05 for comparison between SDF-1-treated cells and GLP-1-pretreated cells before stimulation with SDF-1 at times indicated. # p<0.001 for comparison between SDF-treated cells and untreated cells; n = 12. B: GLP-1(9-36) does not change cAMP-concentration in CD4-positive lymphocytes. CD4-positive lymphocytes were treated with 10 nM GLP-1(9-36), SDF-1 (100 ng/mL) or pretreated with 10 nM GLP-1(9-36) for 30 minutes before stimulation with SDF-1 (100 ng/mL) for times indicated. Bars represent mean±SEM; n = 12; ***p<0.001 compared with untreated cells.

Given that f-actin formation in isolated human CD4-positive lymphocytes is an important mechanism in cell polarisation during cell migration [16], [17], we next investigated the effect of GLP-1(9-36) on f-actin formation in isolated human CD4-positive lymphocytes using flow cytometry analysis. As demonstrated in figure 4A, GLP-1(9-36) significantly reduced SDF-1-induced f-actin formation.

### The Effect of GLP-1(9-36) on Chemokine-induced Migration of Human CD4-positive Lymphocytes is not Mediated by cAMP Signalling

As cAMP has been suggested to be involved in GLP-1(9-36) signalling [18], we performed a quantitative cAMP assay of isolated CD4-postitive lymphocytes in order to investigate whether the effect of GLP-1(9-36) on chemokine-induced CD4-positive lymphocyte migration is caused by cAMP signalling. In this assay forskolin was used as a positive control and resulted in a 5.2-fold increase in cAMP levels (p<0.001). No change of cAMP was observed with GLP-1(9-36) in a time-response analysis (Fig. 4 B). Furthermore, no increase in cAMP was observed when cells were incubated with SDF-1 plus GLP-1(9-36) under the same conditions as in the migration experiments. Therefore, GLP-1(9-36) inhibits chemokine-induced CD4-positive lymphocyte migration independent of cAMP signalling.

### The GLP-1 Receptor does not Mediate the Inhibitory Effect of GLP-1(9-36) on Chemokine-induced CD4-positive Lymphocyte Migration

**Figure 5:**
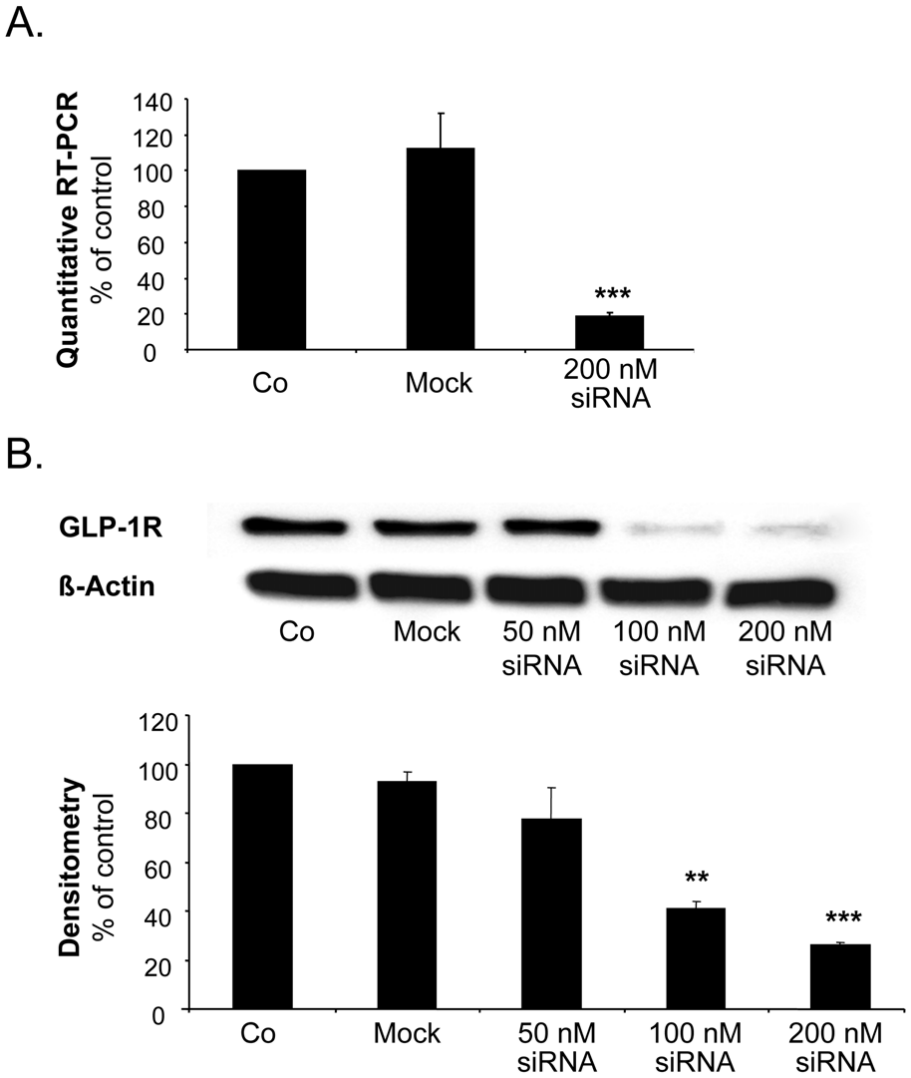
Transfection of isolated human CD4-positive lymphocytes with GLP-1 receptor siRNA reduces both GLP-1 receptor mRNA and protein levels. A. and B.: Transfection with 200 nM GLP-1R siRNA effectively reduces the amount of GLP-1 receptor mRNA (A.) and protein (B.) levels in isolated human CD4-positive lymphocytes. Gene expression (A.) and a representative Western blot are depicted together with quantitative analysis using densitometry (B.). Quantitative analysis for both GLP-1 receptor mRNA (A.) and protein levels (B.) are displayed as mean±SEM; n = 4 for (A.) and n = 5 for (B.); **p<0.01, ***p<0.001 compared to controls.

**Figure 6:**
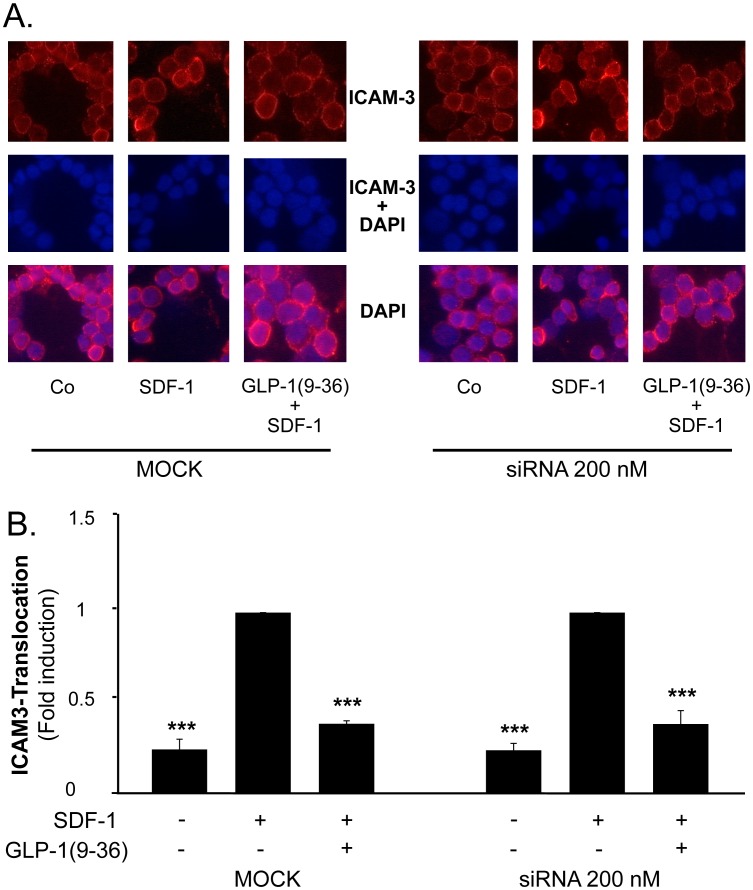
GLP-1(9-36) reduces SDF-1-induced ICAM3 translocation in GLP-1R siRNA transfected human CD4-positive lymphocytes. A: MOCK and siRNA transfected CD4-positive lymphocytes were pretreated with 10 nM GLP-1(9-36) for 30 min before stimulation with SDF-1 for 30 min. ICAM3 translocation was determined using immunofluorescence staining as described in figure 4. B: Statistical analysis of cells positive for ICAM3 translocation are presented as fraction of polarized cells; bars represent mean±SEM; n = 5; ***p<0.001 compared with SDF-1-stimulated cells.

As binding to the GLP-1 receptor protein has been shown to reduce CD4-positive lymphocyte migration [9], we investigated if GLP-1(9-36) reduces T-cell migration via the known GLP-1 receptor. Transfection with siRNA resulted in a markedly reduced expression of both GLP-1 receptor mRNA and protein levels in isolated human CD4-positive lymphocytes (figure 5A+B). As demonstrated in figure 6A+B, GLP-1(9-36) significantly inhibited SDF-1 induced ICAM3 translocation in both GLP-1 receptor positive and negative human CD4-positive lymphocytes, suggesting an effect independent of the known GLP-1 receptor.

## Discussion

This study demonstrates that the degradation product GLP-1(9-36) inhibits chemokine-induced migration of human CD4-positive lymphocytes, which is mediated through an early inhibition of PI-3 kinase activity with subsequent inhibition of MLC and cofilin phosphorylation as well as f-actin formation and ICAM3 translocation. The action of GLP-1(9-36) on CD4-positive lymphocyte migration and the PI3-kinase signaling cascade is not mediated by cAMP signaling or the GLP-1 receptor protein.

Current new treatment options for patients with type 2 diabetes include DPP-IV inhibitors, which decrease the rapid degradation of GLP-1(7-36) to the metabolite GLP-1(9-36). Given that patients with type 2 diabetes are a high-risk population for cardiovascular events [3], beneficial vascular effects of the metabolite GLP-1(9-36) on the cardiovascular system are therefore of potential clinical interest. Recently, a role of GLP-1(9-36) in preventing ischemia-reperfusion injury has been suggested in isolated perfused mouse hearts [6]. Nikolaidis et al. showed that the degradation product GLP-1(9-36) improves left ventricular and systemic hemodynamics using a canine model of dilated cardiomyopathy [7]. However, nothing is known about the effects of GLP-1(9-36) on CD4-positive lymphocyte migration, an early and critical step in atherogenesis [8]. Our study extends the current knowledge on GLP-1(9-36) by demonstrating an inhibition of chemokine-induced CD4-positive lymphocyte migration, an effect which could potentially contribute to the modulation of vascular inflammation in diabetic patients.

The effect of GLP-1(9-36) on human CD4-positive lymphocyte migration is mediated by an early inhibition of PI-3 kinase activity, an important step in chemokine-induced human CD4-positive lymphocyte migration. PI-3 kinase activity results in the activation of small Rho GTPases. Downstream of these GTPases, the phosphorylation of cofilin is important to allow for actin polymerization of migrating cells, while the phosphorylation of MLC contributes to cell contraction at the uropod of migrating T-cells. In this study, GLP-1(9-36) reduces chemokine-induced phosphorylation of cofilin with subsequent f-actin formation. Moreover, GLP-1(9-36) limits the phosphorylation of MLC and inhibits SDF-1-mediated ICAM3 translocation to the uropod of migrating cells, thus inhibiting two crucial steps in T-cell migration.

GLP-1(9-36) diminishes both SDF-1- as well as RANTES-induced lymphocyte migration and thus the effect of GLP-1(9-36) on cell migration does not appear to be depended on the chemotactic stimulus employed. However, the concentrations of GLP-1(9-36) necessary to inhibit T-cell migration were physiological for RANTES, but supraphysiological for SDF. This may be caused by the fact that SDF was observed to be a much stronger inducer for T-cell migration (3.43-fold for SDF vs 1.98-fold for RANTES), with higher GLP-1(9-36) concentrations necessary to inhibit SDF compared to RANTES - induced T-cell migration.

Moreover, the GLP-1(9-36) concentrations employed in our study correspond to the concentrations used in previous experiments [7], [19] and the effects observed are not caused by alterations in cell viability or the chemokine receptor expression. Furthermore, the phenomenon demonstrated is not due to unspecific effects of the peptide nor caused by endotoxins as shown by experiments with scrambled GLP-1(9-36) and heat-inactivated GLP-1(9-36). The effect of GLP-1(9-36) on chemokine-induced CD4-positive lymphocyte migration and the PI-3 kinase signaling cascade was not mediated by intracellular levels of cyclic AMP, which has previously been suggested to be involved in GLP-1(9-36) signaling [18]. Thus, signaling in isolated human CD4-positive lymphocytes is therefore different from rodent neonatal cardiomyocytes, in which GLP-1(9-36) results in increased levels of cAMP and increased phosphorylation of the PI-3 kinase target Akt [18]. Furthermore, we could demonstrate that the inhibitory action of the degradation product GLP-1(9-36) on CD4-positive lymphocyte migration is independent of the GLP-1 receptor protein, which has previously been shown to mediate the inhibitory effect of untruncated GLP-1 on chemokine-induced CD4-positive lymphocyte migration [9].

Potential mechanisms by which GLP-1(9-36) may inhibit PI3-kinase activity may be at the level of the chemokine receptor via interaction with a so far unknown GLP-1(9-36) receptor, mediated by G-proteins, caused by dimerization of and with PI3-kinase or via modification of the regulatory p85 or catalytic p110 subunit.

Thus, the present study shows that GLP-1(9-36) inhibits chemokine-induced CD4-positive lymphocyte migration by inhibition of the PI3-kinase pathway independent of cAMP and GLP-1 receptor signaling. Further studies are needed to assess whether such effects may be clinically relevant for type 2 diabetes patients treated with DPP-IV inhibitors.
