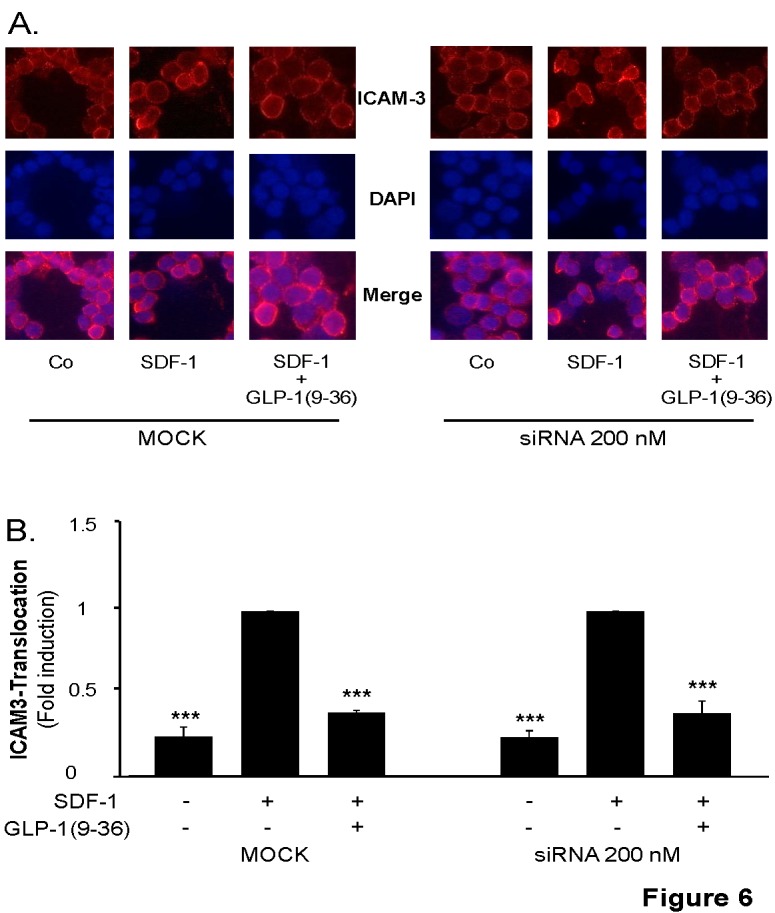# Correction: Glucagon-Like Peptide-1(9-36) Inhibits Chemokine-Induced Migration of Human CD4-Positive Lymphocytes

**DOI:** 10.1371/annotation/cdb46efb-fa7c-4507-93e9-eb739804ed8f

**Published:** 2013-10-16

**Authors:** Ana Liberman, Melanie Esser, Nikolaus Marx, Mathias Burgmaier

In Figure 6 section A, the second row should be labeled "DAPI" instead of "ICAM-3+DAPI" and the third row should be labeled "Merge" instead of "DAPI."

Please see the corrected Figure 6 here: 

**Figure:**